# Lactate induces oxidative stress by HIF1α stabilization and circadian clock disturbance in mammary gland of dairy cows

**DOI:** 10.1186/s40104-025-01181-1

**Published:** 2025-05-01

**Authors:** Juan Feng, Lei Zhu, Cunman He, Ruidong Xiang, Jianxin Liu, Jie Cai, Diming Wang

**Affiliations:** 1 Institute of Dairy Science, College of Animal Sciences, Zhejiang University, Hangzhou, 310058 People’s Republic of China; 2 College of Life Science, Zhejiang University, Hangzhou, 310058 People’s Republic of China; 3 Agriculture Victoria Research, AgriBio Centre for AgriBioscience, Bundoora, VIC, 3052 Australia; 4 School of Applied Systems Biology, La Trobe University, Bundoora, VIC 3052 Australia; 5 Department of Veterinary Medicine, College of Animal Sciences, Zhejiang University, Hangzhou, 310058 People’s Republic of China

**Keywords:** Circadian, HIF1α, Lactate, Mammary gland, Oxidative stress

## Abstract

**Background:**

Lactate is a classical byproduct of glucose metabolism, and the main lactate production pathway depends on glycolysis. Lactate stabilized HIF1α by inhibiting PHD activity, leading to hypoxic stress response and exacerbating glycolysis in multiple tissues. However, the redox induction mechanism of lactate in mammary gland has not been understood yet. Herein, we describe a lactate-responsive HIF1α/circadian control mechanism in oxidative stress in the mammary glands of dairy cows.

**Results:**

The in vivo study showed that dairy cows with high lactate concentrations are associated with reduced milk yield and more ROS accumulation in mammary gland. Western blot results in MAC-T cells showed positive correlation between lactate concentrations, expression of HIF1α and oxidative stress indicators, but not circadian core components. To test how lactate-mediated HIF1α dysfunction leads to cell protection process, we investigated altered expression of circadian core related genes following HIF1α stabilization. We found that stabilized HIF1α by lactate inhibited stimulated expression of circadian core components due to the similarity of HRE and E-box transcription elements. Furthermore, we found that lactate treatment strengthened the binding of *HIF1α* with *BMAL1*, *HMOX1* and *FOXO3* in MAC-T cells. Moreover, *HIF1α* knockdown altered expression of circadian rhythm related genes and reduced oxidative stress state.

**Conclusion:**

In summary, our study highlights the central role of competitive transcriptional element occupancy in lactate-mediated oxidative stress of mammary gland, which is caused by HIF1α stabilization and circadian rhythm dysfunction. Our findings introduce a novel nutritional strategy with potential applications in dairy farming for optimizing milk production and maintaining mammary gland health.

**Supplementary Information:**

The online version contains supplementary material available at 10.1186/s40104-025-01181-1.

## Introduction

Metabolites play crucial roles in various cellular function regulation processes, including cell proliferation, differentiation, stress response, and apoptosis. Lactate is the final product of glycolysis in many organisms under anaerobic conditions [1]. Contrary to its early portrayal as a metabolic waste product and a fatigue agent, lactate serves as the principal messenger in a complex feedback loop system [2], and has been shown to signal through its specific receptor G protein-coupled receptor 81 (GPR81) [3] or to be transported into cells by monocarboxylate transporters (MCTs) [4]. Recently, lactate is reported to be involved in reactive oxygen species (ROS) production [3]. Lactate influences cell and tissue function through mechanisms such as allosteric binding, and chromatin reprogramming via histone lysine modification [5]. However, the role of lactate plays in redox status and phenotype regulation in farm animals are remained to be investigated.

In recent decade, lactate has been known to be involved in oxygen metabolism in various of tissues. Under normoxic conditions, hypoxia inducible factor 1 subunit alpha (HIF1α) undergoes post-translational modification by prolyl-hydroxylases and is targeted for proteasomal degradation by the Von Hippel-Lindau (VHL) E3 ubiquitin ligase. Conversely, under hypoxic conditions or during mitochondrial stress, HIF1α subunits are shielded from degradation due to the inactivation of oxygen-dependent prolyl-hydroxylases [6]. Inhibition of PHD activity by lactate stabilizes HIF1α, leading to exacerbation of glycolysis [7]. A recent study found that lactate can upregulate the expression of NDUFA4L2 (NADH dehydrogenase (ubiquinone)-1α subcomplex 4-like 2) through a transcription factor HIF1α mediated mechanism [8]. The information suggested that the stabilized HIF1α/β heterodimer activates gene transcription pathways involved in angiogenesis and anaerobic glycolysis [9].

All mammals possess an internal circadian clock, which couples sleep-wake and fasting-feeding cycles with the light-dark cycle [10, 11]. In mammals, the molecular clock is encoded by a transcription-translation feedback loop composed of activators (CLOCK/BMAL1), which induce the transcription of repressors (PER/CRY) that feedback to inhibit the rising phase in a cycle that repeats itself every 24 h, including an additional stabilizing loop comprised of REV-ERB/ROR transcription factors (TFs) [12]. In addition to light, circadian clock is also affected by various of environmental signals, including ATP/AMP, redox status, and nuclear receptor ligands [13]. For farm animals, milk yield and composition of dairy cow are considered 24 h rhythm pattern [14], and mRNA expression level of *BMAL1* and *CLOCK* genes in mammary gland is higher than in other tissues, indicating potential role of rhythms in coordinating milk synthesis [15]. In humans, it has been found that the activity and GSH concentration of the oxidative stress kinase (CAT, SOD-1, GSH-Px, R-GSSG, GST) in vivo are regulated in circadian rhythm [16]. Understanding nutritional approach in mediating circadian clock is an alternative way in regulating oxidative stress in mammalian animals.

In this study, we attempted to understand the mechanism of lactate in regulating oxidative stress in mammary gland of lactating dairy cows. We found high milk lactate concentration is associated with lower milk yield and more severe oxidative stress in mammary gland of dairy cows in both in vivo and in vitro systems. Moreover, we found lactate-mediated HIF1α stabilization plays key role in dysfunction circadian rhythm in mammary epithelial cells. Moreover, we revealed that shared HREs containing the sequence 5′-[A/G]CGTG-3′ between HIF1α and circadian rhythm (*BMAL1*, *CREB1* and *SERPINE1*) are the cause of increased expression of oxidative related genes (*HMOX1*, *FOXO3* and *CPT1A*) triggered by lactate. Our study suggested that there exist a competitive transcriptional element occupancy mediated-approach in mediating oxidative stress via in mammalian system.

## Materials and methods

### Animal management

All experimental procedures were approved by the Institutional Animal Care and Use Committee at Zhejiang University. Based on a power analysis with high reliability (Table S1), we chose 24 lactating Holstein cows with similar parity (2–3) and DIM (187 ± 5 d) from 60 cows in Hangzhou Zhengxing Dairy Company (Zhejiang, China). Sixteen animals were divided as two groups, high lactate group (*n* = 12, milk lactate > 1.4 mmol/L) and low lactate group (*n* = 12, milk lactate < 1 mmol/L). All selected dairy cows were housed in free-stalls and provided with a total mixed ration primarily consisting of corn silage, sorghum silage, and concentrate supplements to meet their nutritional needs as determined by the National Research Council (NRC) guidelines [17]. The ingredients and chemical compositions of the basal diet were described in details previously [18]. Access to drinking water was provided through automatic water bowls, and milking was conducted three times a day to record milk yield of the individual cows.

### Milk sample collection

Milk yield was measured for 3 consecutive days. Milk samples were collected at 06:30, 10:00 and 19:00 every day, and 50 mL of milk was mixed at a ratio of 4:3:3 to analyse the composition. Milk composition traits (fat, protein, and lactose percentages, and urea concentration) were measured with an FT6000 Milkoscan infrared analyzer (Foss Electric A/S). Somatic cell count (SCC; cells/mL) was determined with a Fossomatic 7 DC analyzer (Foss Electric A/S). Likewise, milk reactive oxygen species were measured by reactive oxygen species assay kit (ROS; S0033S; Beyotime Institute of Biotechnology, Jiangsu, China). In addition, commercial assay kits from Nanjing Jiancheng Bioengineering Institute (Jiangsu, China) were used to quantify concentration of milk malondialdehyde (MDA; A003-1-2), superoxide dismutase (SOD; A001-3-2), total antioxidant capacity (T-AOC; A015-2-1), glutathione peroxidase (GSH-Px; A005-1-2), total nitric oxide synthase (T-NOS; A014-1-2).

### Cell culture and treatment

The MAC-T cells were presented by Professor Hongyun Liu (Zhejiang University, China). The MAC-T cells were cultured in DMEM (Gibco, NY, USA) supplemented with 10% FBS (Invitrogen, Carlsbad, CA, USA) and 1% penicillin/streptomycin (Hyclone, Beijing, China) at 37 °C in a 5% CO_2_ atmosphere. As for oxidative stress related variables, MAC-T cells were seeded into a 6-cm dish at the density of 3.5 × 10^6^ cells/mL and incubated with or without sodium lactate (15 mmol/L, 30 mmol/L) for 24 h and measured by the commercial assay kit mentioned before, all the data were normalized with protein concentration by Enhanced BCA Protein Assay Kit (P0010S; Beyotime Institute of Biotechnology, Jiangsu, China). For synchronization test, MAC-T cells were synchronized with dexamethasone (100 nmol/L, 30 min) and collected at indicated time points.

### Lactate measurements in milk and MAC-T cells

MAC-T cells were seeded into a 6-cm dish at the density of 5 × 10^6^ cells/mL and incubated with or without sodium lactate (15 mmol/L, 30 mmol/L) for 24 h and measured by lactic acid content assay kit (BL868B; Biosharp Biotechnology, Anhui, China), the data were normalized with protein concentration by Enhanced BCA Protein Assay Kit (P0010S; Beyotime Institute of Biotechnology). As for lactate measurements in milk, the milk was centrifuged at 12,000 r/min at 4 °C for 10 min, and the supernatant was detected by lactic acid content assay kit (BL868B; Biosharp Biotechnology).

### SiRNA transfection

We used commercial siRNA products for transient knockdown of *HIF1α*. For RNA interference, the MAC-T cells cultured in six-well plates were transfected with siHIF1α or control (siNC), respectively, using riboFECT CP Transfection Kit (166 T) according to the manufacturer’s protocol (C10511-05; Guangzhou Ribo Biotechnology, Guangdong, China). MAC-T cells were then plated at a density of 1 × 10^6^ per well; optimal transfection time was of 48 h and 72 h as the maximal silence efficiency evidenced by qPCR and western blot analysis. Otherwise, siRNAs were synthesized from Hangzhou Rui Pu biological Technology (China). The sequences of siRNAs are listed in Table S2.

### RNA-sequencing analysis

Total RNA from the MAC-T cells was extracted with TRIzol reagent according to the manufacturer's instructions (Aidlab Biotechnologies Co., Ltd., Beijing, China; Code: RN03). Assessment of RNA integrity was performed using the RNA Nano 6000 Assay Kit on the Bioanalyzer 2100 system (Agilent Technologies, CA, USA). The sequencing procedure was performed according to our previously method [19]. Before conducting the analysis of differential gene expression, read counts from each library were adjusted using the edgeR program package by applying one scaling normalized factor. The differential expression analysis between two conditions was carried out using the edgeR package (version 3.22.5). The *P*-values were adjusted using the Benjamini & Hochberg method. A corrected *P*-value threshold of 0.05 and an absolute fold change threshold of 2 were set to determine significantly differential expression. The WebGestalt was utilized for Gene Ontology (GO) enrichment analysis and KEGG analysis of differentially expressed genes [20]. GO terms and KEGG pathway that had a corrected *P*-value of less than 0.05 were considered significantly enriched by the differentially expressed genes. The analysis of rhythmic expression with eJTK_Cycle (v3.1.R) were according to previous studies [21]. Adjusted *P*-values of 0.1 and 0.9 were chosen for ‘rhythmic’ and ‘non-rhythmic’ genes respectively. Genes were classified as “unaffected” if they were similarly rhythmic in both conditions, as “gain” if they were non-rhythmic in the control condition and rhythmic in the experimental condition, as “loss” if they were rhythmic in the control condition and non-rhythmic in the experimental condition, and “phase-shift” if they were rhythmic in both conditions but were annotated with peak phases that were greater than 4 h different. Heatmaps were sorted by peak phase for the control condition for loss, unaffected, and phase-shift reprogramming groups, while they were sorted by peak phase of the experimental condition for the gain reprogramming group.

### RNA extraction and qPCR

RNA was extracted from cell samples using FastPure Cell/Tissue Total RNA Isolation Kit V2 (RC112-01; Nanjing Vazyme Institute of Biotechnology, Jiangsu, China) according to the manufacturer’s instructions. cDNA was synthesized from 1 μg total RNA using HiScript II Q RT SuperMix for qPCR (+ gDNA wiper) (R223-01; Nanjing Vazyme Institute of Biotechnology). Quantitative real-time PCR (qPCR) was performed according to the manufacturer’s instructions using a kit (AceQ qPCR SYBR Green Master Mix (Low ROX Premixed); Q131-02; Nanjing Vazyme Institute of Biotechnology). All qPCR reactions were performed in an ABI7500 (Thermofisher, Waltham, MA, USA) sequence detector. The primer sequences shown in the Table S2. The mRNA levels were normalized to *GAPDH* mRNA and expressed as fold change relative to the control group.

### Luciferase analysis

For the luciferase assay, the cells cultured in 96-well plates at 80%–90% confluence were cotransfected with 100 ng of the vector (HRE luciferase) plus 10 ng of control vector (Renilla luciferase) per well using Lipofactamine 3000 reagent (Invitrogen, USA). The Dual-Luciferase Reporter Assay Kit (DL101-01, Nanjing Vazyme Institute of Biotechnology) was used to measure luciferase activity on a Tecan SPARK microplate reader (Tecan, Männedorf, Switzerland) according to the kit manufacturer’s protocol. The relative luciferase activity was calculated as the ratio of firefly luciferase compared with Renilla luciferase activity.

### Western blotting

Cell lysates were prepared in ice-cold RIPA buffer containing protease inhibitor. The cells were lysated on ice for 30 min and vortexed every 10 min. Lysates were centrifuged at 13,000 × *g* for 10 min at 4 °C. Part of the supernatant was taken to determine the protein concentrations using the Enhanced BCA Protein Assay Kit (P0012S; Beyotime Institute of Biotechnology). A total of 10 μg of protein from each sample was separated by 8% SDS-PAGE. The molecular location of the bovine target protein on the gel aligned with the manufacturer’s specified antibody statement regarding the molecular weight of the target protein, as indicated by the molecular weight marker (26616; Thermo Fisher Scientific, Waltham, MA, USA). The target protein on gel was electrophoretically transferred to a polyvinylidene difluoride membrane (PVDF). The membranes were blocked in Western blocking buffer (P0023B; Beyotime Institute of Biotechnology) at room temperature for 1 h. The membranes were incubated overnight with the following primary antibodies against: HIF1α (1:1,000; ab228649; Abcam, Cambridge, MA, USA), CLOCK (1:1,000; 5157S; Cell Signaling Technology, Danvers, MA, USA), BMAL1 (1:1,000; ab235577; Abcam), FOXO3 (1:1,000; A9270; Abclonal Technology), HMOX1 (1:1,000; A19062; Abclonal Technology) and β-actin (1:2,000; ABP57456; Abbkine Scientific, Atlanta, GA, USA). Subsequently, the PVDF membranes were washed with Tris-buffered saline/Tween (TBS-T) 3 times and probed with the appropriate horseradish peroxidase (HRP)-conjugated secondary antibodies (1:5,000; ab6721, Abcam) at room temperature for 1 h. Immunoreactive bands were visualized by SuperFemto ECL Chemiluminescence Kit (E423-01; Nanjing Vazyme Institute of Biotechnology). β-Actin was used as a reference protein in this study. Each experiment was repeated at least 3 times. The signals were quantified using the ChemiDoc Imaging System by Biorad. Lastly, all Western blot bands were analyzed using Image-J (Media Cybernetics, Rockville, MD, USA).

### Chromatin immunoprecipitation-quantitative RT-PCR (ChIP-qPCR)

Chromatin immunoprecipitation was performed using NovoNGS CUT&Tag 3.0 High-Sensitivity Kit (N259-YH01; Novoprotein, Jiangsu, China) with an antibody specific for HIF1α (1:100; NB100-105, Novus, Colorado, USA) or normal rabbit IgG (1:100; 3900S, Cell Signaling Technology). Following ChIP, quantitative PCR was utilized to amplify and quantify the immunoprecipitated DNA using primers specific for the *HIF1α* within *BMAL1*, *HMOX1* and *FOXO3* (Table S5).

### Statistical analysis

Sample size was determined according to logistical (power analysis) and financial constraints due to the intense sampling and analytical protocol. All data were tested for normality and homogeneity of variance using the Shapiro–Wilk and Levene tests, respectively. Differences in milk performance traits and oxidative stress related variables between the LL and HL cows were assessed using the independent samples *t*-test. Differences in oxidative stress related variables between the Con, 15LNa and 30LNa group were assessed using the one-way ANOVA (and nonparametric). Data from qPCR were analyzed using the 2^−ΔΔCt^ method and normalized to the respective control (siNC). Data were analyzed with Graphpad Prism (v8) and R software (v4.1.1). Data were presented as mean ± SEM. Statistical significance (exception for omics data as described above) was declared at *P* < 0.05, *P* < 0.01 and at *P* < 0.001.

## Results

### Lactation performance and oxidative stress variables in cows with different milk lactate concentration

**Fig. 1:**
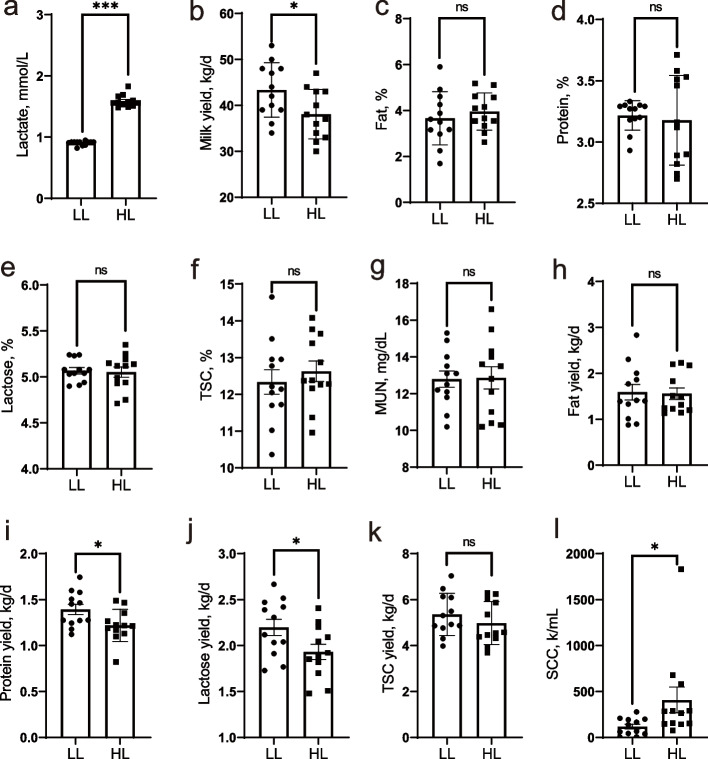
Milk performance in the cows with high lactate (HL) and low lactate (LL). TSC, total solids content; MUN, milk urea nitrogen. *** represents *P* < 0.001, * represents *P* < 0.05, ns represents *P* ≥ 0.1. Error bars indicate the standard error of the mean (SEM). *n* = 12

As shown in Fig. 1, the milk lactate concentration was higher in high lactate (HL) group than in low lactate (LL) group (*P* < 0.01, Fig. 1a). The HL-cows are lower in yields of raw milk, lactose and fat, compared to animals in LL group (*P* < 0.05, Fig. 1b, i and j). Additionally, the high lactate group showed significantly higher somatic cell count than the low SCC group (*P* = 0.04, Fig. 1l). In addition, MDA and T-NOS concentrations were higher in HL-animals compared to LL-animals, whereas the concentration of GSH-Px, SOD and T-AOC were lower in HL-cows than that of LL-ones (Table 1).

**Table 1:** Milk oxidative stress variables in the cows with high lactate (HL) and low lactate (LL)

**Item**^1^	**LL**	**HL**	***P*-value**	**SEM**
GSH-Px, U/mL	726.6^a^	535^b^	< 0.05	88.41
MDA, nmol/mL	3.92^b^	15.62^a^	< 0.05	5.46
SOD, U/mL	28.28^a^	17.94^b^	< 0.01	2.97
T-AOC, mmol/L	1.661^a^	1.621^b^	< 0.05	0.0181
T-NOS, U/mL	27.79^b^	52.26^a^	< 0.01	4.25

*GSH-Px* Glutathione peroxidase, *MDA* Malondialdehyde, *SOD* Superoxide dismutase, *T-AOC* Total antioxidant capacity, *T-NOS* Total nitric oxide synthase

^a,b^Means in the same row with different superscripts are statistical different (*P* < 0.05)

### Lactate led to more severe oxidative stress in MAC-T cells

**Fig. 2:**
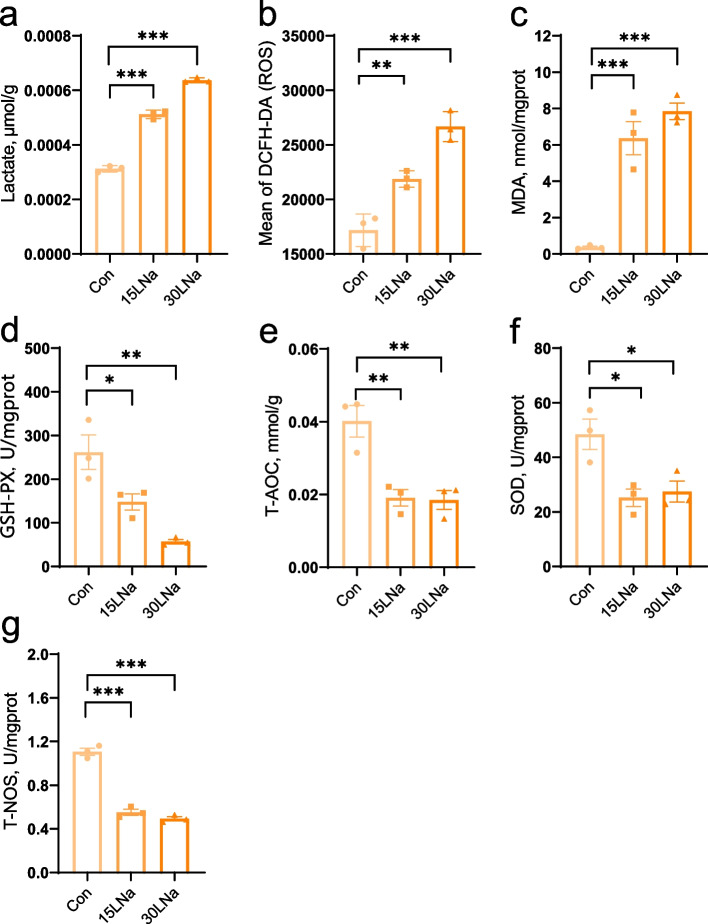
The oxidative stress variables and lactate concentration of MAC-T cells in the control (Con), 15 mmol/L sodium lactate (15LNa) and 30 mmol/L sodium lactate (30LNa) group. ROS, reactive oxygen species; MDA, malondialdehyde; GSH-Px, glutathione peroxidase; T-AOC, total antioxidant capacity; SOD, superoxide dismutase; T-NOS, total nitric oxide synthase. *** represents *P* < 0.001, ** represents *P* < 0.01, * represents *P* < 0.05. Error bars indicate the standard error of the mean (SEM). *n* = 3

Following treating MAC-T cells with different dose of sodium lactate (15 mmol/L sodium lactate (15LNa) and 30 mmol/L sodium lactate (30LNa)) measured the lactate concentration in MAC-T cells (Fig. 2a), we found ROS and MDA were significantly higher in cells treated with 15LNa and 30LNa group compared to the control group (*P* < 0.01, Fig. 2b and c). In contrast, GSH-Px, T-AOC, SOD and T-NOS were significantly lower in 15LNa and 30LNa treated cells compared to control group (*P* < 0.05, Fig. 2d and g).

### Lactate altered oxidative stress via circadian rhythm and HIF1 signaling pathway in MAC-T cells

**Fig. 3:**
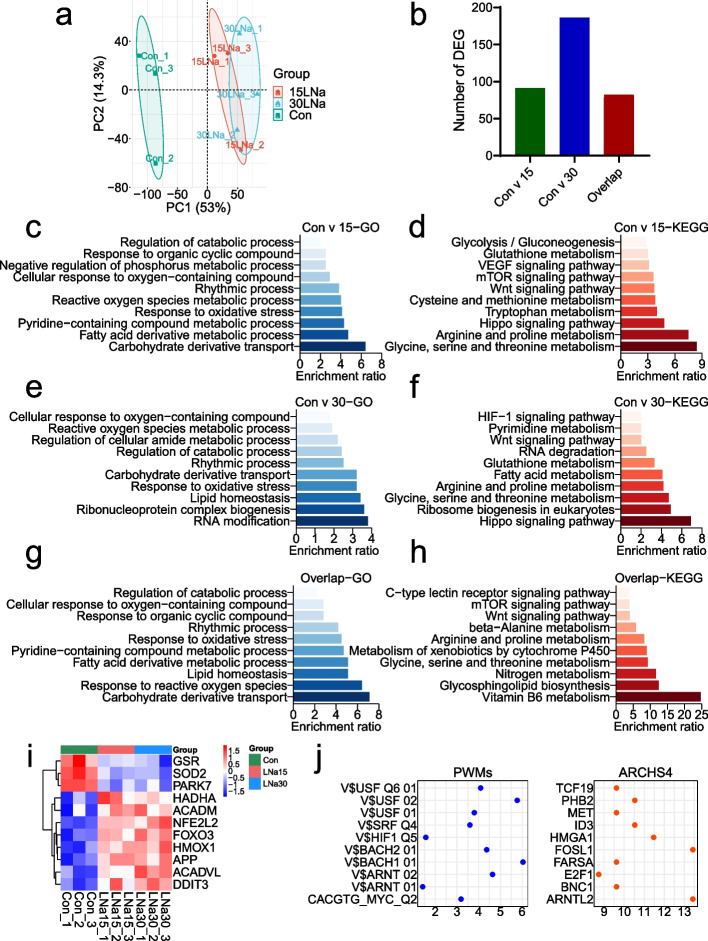
Transcriptional programs of MAC-T cells in the control (Con), 15 mmol/L sodium lactate (15LNa) and 30 mmol/L sodium lactate (30LNa) groups. RNA-seq was performed on freshly isolated milk cells. Significance was determined based on |log_2_Fold Change| > 1 and FDR (False Discovery Rate) < 0.05 using the edgeR algorithm. **a** The PCA analysis of MAC-T cells with Con, 15LNa and 30LNa group. **b** Number of differentially expressed genes (DEG), Con v 15 and Con v 30 DEGs were determined based on |log_2_Fold Change| > 1 and FDR (False Discovery Rate) < 0.05 using the edgeR algorithm and overlap DEGs were the intersection of two groups of Con v 15 and Con v 30. **c–d** KEGG and GO pathway enrichment analysis of DEG between Con and 15LNa group. **e–f** KEGG and GO pathway enrichment analysis of DEG between Con and 30LNa group. **g–h** KEGG and GO pathway enrichment analysis of overlap DEG between the above two groups. **i** Heatmaps and clustering analysis of oxidative stress related genes. **j** The Enrichr analysis of overlap DEG between the above two groups. *n* = 3

The PCA analysis of cells in 15LNa, 30LNa and Con group showed that transcriptome expression profiles of 15LNa and 30LNa group were distinctly separated from those of the control (Fig. 3a). Different numbers of differential expression genes (DEGs) were detected across different treatments: 91 between the control and 15LNa group (C15L), 186 between the control and 30LNa group (C30L) (Fig. 3b). In relative to the control, GO and KEGG analysis of C15L group mainly participate in the response to oxidative stress, reactive oxygen species metabolic process, rhythmic process, Glutathione metabolism and VEGF signaling pathway (Fig. 3c and d). Similarly, DEGs between C30L and Con groups are concerned with response to oxidative stress, reactive oxygen species metabolic process, rhythmic process, glutathione metabolism and HIF-1 signaling pathway (Fig. 3e and f). Compared with the control, functions of shared DEGs between C15L and C30L groups include cellular response to oxygen-containing compound, response to oxidative stress, reactive oxygen species metabolic process and rhythmic process pathway (Fig. 3g and h). To learn genes concerned with oxidative stress by using heat map technique, we found higher expression of oxidative genes (*HMOX1*, *FOXO3*, and *NFE2L2*) in 15LNa and 30LNa group, and lower expression of antioxidative genes (*GSR*, *SOD2*, *PARK7*) in 15LNa and 30LNa group (Fig. 3i). The Enrichr analysis results showed shared DEGs mainly involves HIF1α and BMAL1 transcription factors (Fig. 3j), which is consistent with outcomes observed in KEGG and GO analysis.

**Fig. 4:**
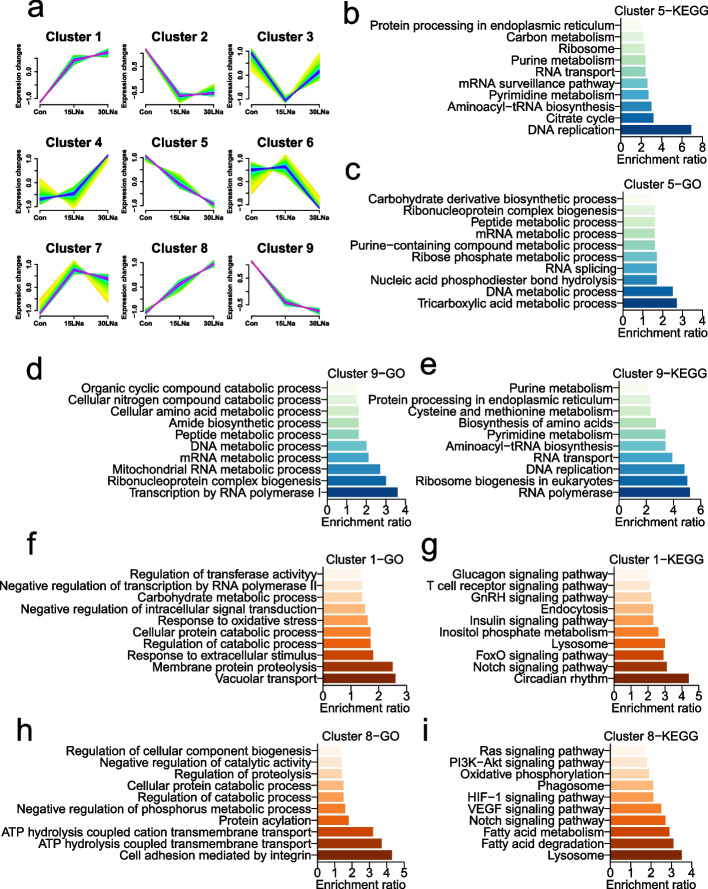
Cluster analysis of differential gene expression in MAC-T cells. **a** Soft clustering analysis from differential genes of all the comparison groups, where each line represents the expression change of one gene. Purple/blue color indicates stronger membership, and green/yellow color denotes weaker membership. **b–c** KEGG and GO pathway enrichment analysis from cluster 5. **d–e** KEGG and GO pathway enrichment analysis from cluster 9. **f–g** KEGG and GO pathway enrichment analysis from cluster 1. **h**–**i** KEGG and GO pathway enrichment analysis from cluster 8. *n* = 3

To examine the dynamic expressional alteration of DEGs under different sodium lactate degrees, we introduced Mfuzz package, and found that 257 DEGs were sorted into 9 clusters, including 2 up-regulated clusters (clusters 1 and 8), 2 down-regulated clusters (cluster 5 and 9) and 5 other mixed clusters (Fig. 4a). The function of up-regulated clusters included response to oxidative stress, circadian rhythm, HIF1 signaling pathway, PI3K-Akt signaling pathway and VEGF signaling pathway, (cluster 1 and 8, Fig. 4f and i). In contrast, functions of down-regulated clusters concerned with DNA replication, RNA transport, DNA metabolic process and mRNA metabolic process pathway (cluster 5 and 9, Fig. 4b and e).

### Lactate disturbed mammary gland circadian and HIF1α stabilization

**Fig. 5:**
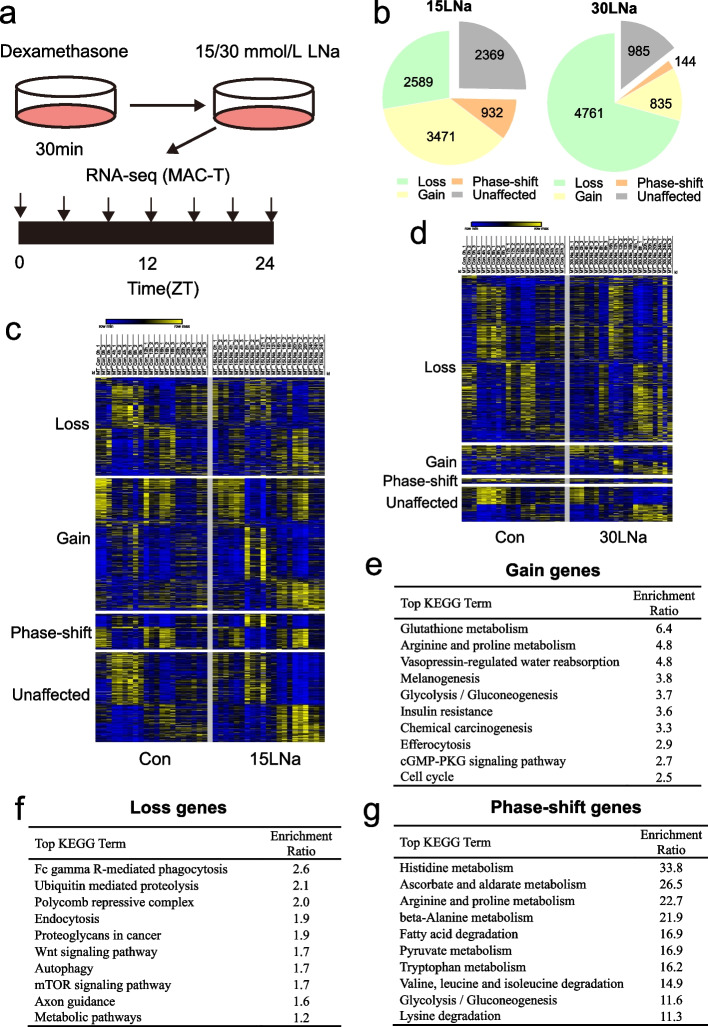
Sodium lactate disrupted the mammary gland circadian transcriptome. **a** MAC-T cells were synchronized with dexamethasone (100 nmol/L, 30 min) and collected at indicated timepoints (*n* = 3/timepoint/group). *ZT* Zeitgeber time. **b** eJTK_Cycle analyses following RNA-seq every 4 h across 24 h identified transcripts that lost, gained, or phase-shift > 4 h oscillations following sodium lactate (FDR-adjusted *P*-value < 0.1 and > 0.9 for cycling and non-cycling genes, respectively). **c** Heatmap of lost, gained, or phase-shift transcripts between Con and 15LNa group. **d** Heatmap of lost, gained, or phase-shift transcripts between Con and 30LNa group. **e** KEGG ontology terms are shown for gain genes overlap between 15 and 30LNa group. **f** KEGG ontology terms are shown for loss genes overlap between 15 and 30LNa group. **g** KEGG ontology terms are shown for phase-shift genes overlap between 15 and 30LNa group

To examine whether lactate affects genome-wide circadian transcription, after treating dexamethasone for 30 min and then sodium lactate for 24 h, cells were harvested for RNA-seq across six time points (Fig. 5a). We identified three major categories of genes following sodium lactate supplementation (Fig. 5b), including those exhibiting (i) a loss (15LNa: 2,589 genes; 30LNa: 4,761 genes), (ii) a gain (15LNa: 3,471 genes; 30LNa: 835 genes), or (iii) a phase-shift in oscillation (15LNa: 932 genes; 30LNa: 144 genes) (FDR-adjusted *P*-values < 0.1 or > 0.9 for oscillating or non-oscillating genes, respectively). Analysis of rhythmic transcription using eJTK_Cycle revealed that reprogramming of 75% of the oscillating transcripts in the cells of 15LNa group relative to the control (Fig. 5c; Table S3), and cells treated with 30LNa group had 85% the oscillating transcripts relative to the control group (Fig. 5d; Table S4).

We overlapped the gain, loss and phase-shift genes of 15LNa and 30LNa groups, respectively. The KEGG analysis of genes that gain oscillations revealed enrichment in pathways associated with Glutathione metabolism, Arginine and proline metabolism and Glycolysis/Gluconeogenesis (Fig. 5e). The KEGG analysis of loss genes enriched in Fc gamma R-mediated phagocytosis, Ubiquitin mediated proteolysis and Polycomb repressive complex pathway (Fig. 5f). We also found the KEGG analysis of genes that phase-shift oscillations enriched in Histidine metabolism, Ascorbate and aldarate metabolism and Arginine and proline metabolism (Fig. 5g).

**Fig. 6:**
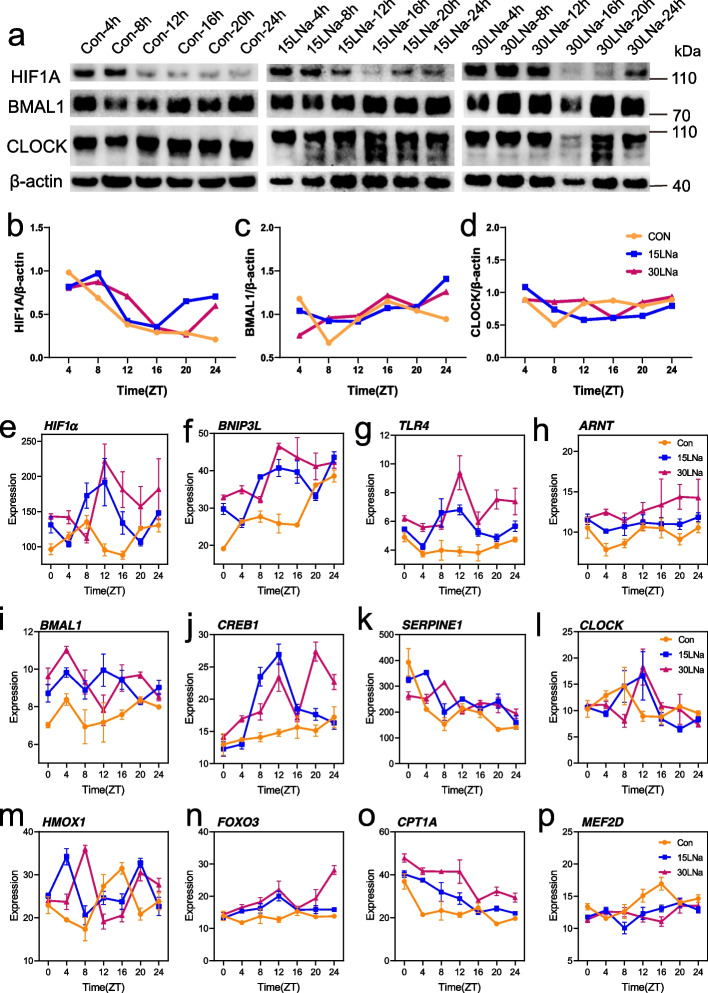
Lactate stabilize the expression of HIF1α. **a** HIF1α, BMAL1 and CLOCK protein levels in MAC-T cells within or without sodium lactate across six time points. **b–d** Quantification of HIF1α, BMAL1 and CLOCK in MAC-T cells within or without sodium lactate across six time points. **e–h** HIF1 pathway genes expression levels in MAC-T cells within or without sodium lactate across six time points. **i–l** Circadian related genes expression levels in MAC-T cells within or without sodium lactate across six time points. **m–p** Oxidative stress related genes expression levels in MAC-T cells within or without sodium lactate across six time points. *ZT* Zeitgeber time

In support of a physical interaction between HIF1α and BMAL1, previous biochemical studies indicated that dimerization between bHLH-PAS proteins, as a high degree of sequence- and structure-level similarity exists between HIF1β (also termed ARNT) and the core clock activator BMAL1 (also termed ARNTL-like, Fig. S1). Moreover, high similarity degree in both sequence- and structure-level were observed between HIF1α and CLOCK (Fig. S2). Then, we evaluated the protein level of HIF1α, BMAL1 and CLOCK across six time points within 24 h, we found that the expression level of HIF1α in 15LNa and 30LNa group was higher than that of the control, while the expression level of BMAL1 and CLOCK were not increased treated with lactate (Fig. 6a–d).

### Stabilized HIF1α binding to HRE elements leads to circadian rhythm dysfunction and oxidative stress

**Fig. 7:**
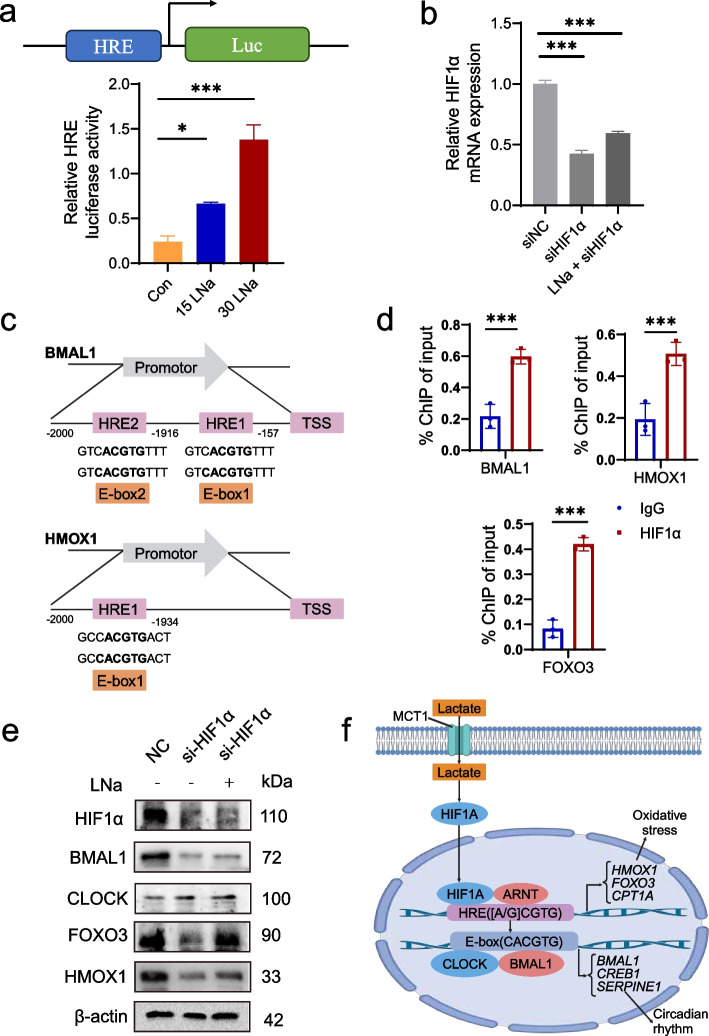
Stabilized HIF1α binds to the HRE sequence to regulate oxidative stress and circadian. **a** Luciferase activity driven by promoters containing HREs with different concentration of sodium lactate. **b** The knock-down efficiency of *HIF1α* was verified by qPCR. **c** The promoter of oxidative stress and circadian related genes contain HRE elements. **d** ChIP-qPCR analysis of *BMAL1*, *HMOX1* and *FOXO3* binding to HIF1α promoter. n = 3 per condition. **e** The protein levels of BMAL1, CLOCK, HMOX1 and FOXO3 after knocking down HIF1α. **f** Graphical abstract of lactate induced oxidative stress of mammary gland and disturbance of mammary gland circadian by stabilizing HIF1α

In the time-series analysis, downstream molecules of HIF1 pathway (*HIF1A*, *BNIP3L*, *TLR4* and *ARNT*) were increased in 15LNa and 30LNa group compared with the control (Fig. 6e–h). These molecules are associated with circadian rhythm (*BMAL1*, *CREB1* and *SERPINE1*; Fig. 6i, j and k) and oxidative stress (*HMOX1*, *FOXO3* and *CPT1A*; Fig. 6m, n and o) were also augmented in 15LNa and 30LNa group than that of the control. However, the expression level of circadian related gene (*CLOCK*) and oxidative related gene (*MEF2D*) are not higher in 15LNa and 30LNa treated cells compared with the control (Fig. 6l and p). We found stabilized HIF1α binds to the HRE elements and enhance the luciferase reporting activity of HRE (Fig. 7a). We also interestingly found that the promoter region of circadian associated gene (*BMAL1*) had HRE element, and the oxidative stress related gene (*CPT1A*) have HRE and E-box element (Fig. 7c). Furthermore, we confirmed that *BMAL1*, *HMOX1* and *FOXO3* binds to the *HIF1α* promoter in sodium lactate treated MAC-T cells (Fig. 7d). However, the promoter region of *CLOCK* and *MEF2D* neither had HRE response nor E-box element (Fig. 7c). Consistent with the mRNA level, the protein abundance of HIF1α and BMAL1 reduced markedly following *HIF1α* knockdown (Fig. 7b and e), and the protein level of oxidative stress and circadian rhythm related genes were increased in cells treated with sodium lactate compared with the control, after *HIF1α* is knocked-down (Fig. 7e).

## Discussion

Lactate plays an important role in regulating metabolic homeostasis, regulation of the circadian is also influenced by metabolites. The incidence of mammary gland health issues and milk performance are associated with changes of lactate status in mammary gland of dairy cows. Assessing the mechanism of lactate induced oxidative stress would offer new opportunity for interpreting mammary gland health status of lactating dairy cows.

The high lactate concentrations in lowered lactation performance and more severe oxidative stress of dairy cows suggested that lactate is one of key cause for metabolic disorders in mammary gland of dairy cows, which has been approved in the in vitro system. This evidence is consistent with our previous study, which suggested excess glucose supply can cause mammary gland glycolysis to enhance and produce large amounts of lactate, thus impaired mammary gland health and reduced its milk performance [22]. Also, other study that lactate was found to promote oxidative stress resistance through hormetic ROS signaling [23].

The predominant enrichment of HIF1 signaling and VEGF signaling pathway in response to lactate treatment is consistent with previous findings that lactate appears to enable the accumulation of HIF1α under aerobic conditions [7, 24, 25]. Furthermore, HIF1α acts as a key transcription regulator mediating a series of genes involved in glycolytic energy metabolism, angiogenesis and cell survival, including vascular endothelial growth factor (VEGF) [26, 27]. The highly enrichment of oxidative stress pathway following lactate treatment is consistent with our previous findings on phenotypic traits. Studies have shown that extracellular factors such as temperature, hormones, and metabolites can also affect the clock and thereby constitute mechanisms for local synchrony of cellular clocks [28], which is agreed with our studies circadian rhythm pathway is predominantly enriched responding to lactate treatment. Moreover, the eJTK_Cycle analysis presented here implicate a role for lactate in remodeling the circadian transcriptome, and is agreed with previous studies that intermediate products of nutrient metabolism give rise to several small molecules that can affect clock function [29]. Predominant enrichment of glutathione metabolism in overlap gain oscillations genes of 15LNa and 30LNa treated cells, suggested oxidative stress related gene may be rhythmic by sodium lactate treatment.

Interestingly, we found that lactate enable to accumulate the expression of HIF1α while the proteins that are the core components of the circadian (BMAL1 and CLOCK) have not been affected by sodium lactate treatment. However, the level of transcriptional of the core components of circadian clock (*BMAL1*, *CREB1* and *SERPINE1*) are increased within sodium lactate treatment. When HIF1α is stable, it translocated to the nucleus and interacts with HRE which contains the sequence 5′-[A/G]CGTG-3′ and activates target gene expression involved in vascularization, glucose transport, energy metabolism and cell migration, to adapt to low oxygen conditions [30, 31]. This E-box-like HRE element happens to be contained in the E-box sequence (5′-CACGTG-3′) regulated by the circadian clock [32]. Therefore, sodiium lactate-stabilized HIF1α may interfere with the transcriptional regulation function of circadian clock by occupying transcriptional elements, leading to abnormal expression mode of circadian clock in mammary gland region.

We found that HRE luciferase activity is enhanced by lactate treatment, which is conducted by stabilizing HIF1α entered into nucleus and binds to HRE [30] and further altered its downstream hypoxic (*HIF1α*, *BNIP3L*, *TLR4* and *ARNT*) and oxidative stress related genes, with HRE elements as their promotors (*HMOX1*, *FOXO3* and *CPT1A*). To investigate the oxidative stress related genes are regulated not only by HIF1α but also by circadian clock. We extracted the promoter of oxidative stress genes, and found the promoters of these genes (*HMOX1*, *FOXO3* and *CPT1A*) are shared by both HIF1α and CLOCK/BMAL1. In other words, since both HIF1 and molecular circadian clocks belong to the bHLH-PAS transcription factor superfamily, due to the similar structure between HIF1α/HIFβ and CLOCK/BMAL1 [33], and leading circadian clock factor BMAL1 forming heterodimer with HIF1α, which finally lead to more severe oxidative stress [34]. The causal effect can be validated that *HIF1α* knockdown reloaded circadian core genes and reduced oxidative stress status in mammary gland. By validating the observation with ChIP-qPCR assay, we confirmed the dominant role of lactate in HIF1α stabilization-mediated in circadian clock disrupting and oxidative stress induction in mammary gland.

The studies presented here reveal lactate induced oxidative stress of mammary gland and disturbance of mammary gland clock by stabilizing HIF1α that may have broader implications for maintaining mammary gland health of lactating ruminants and optimizing the time node of nutrition or disease intervention. We provide evidence for molecular mechanism of stabilizing HIF1α in lactate affecting oxidative stress and circadian transcriptional oscillation. Our study suggested a competitive transcriptional element occupancy-mediated oxidative status regulation mechanism in mammalian system. Moreover, targeted regulation of HIF1α/BMAL1 are potential nutritional approach in reducing metabolic disorders in various mammary gland disorders in dairy animals.

## Conclusion

The current research investigated mechanism of lactate-induced oxidative stress in bovine mammary gland. We found accompany of HIF1 signaling, oxidative stress signaling, and circadian rhythm pathway in responding to lactate treatment. The translational stabilization of HIF1α induced by lactate are cause of dysfunction of CLOCK/BMAL1 mediated circadian network, due to the shared HRE sequences on promoters of both HIF1α and BMAL1 and *HIF1α* knockdown can reduced oxidative stress state by re-stabilization of circadian rhythm network. Our study revealed a unique oxidative stress regulation approach may through competitive transcriptional element occupancy by functional genes, and providing a novel nutritional route to solve mammary gland disorder caused by over accumulation of lactate.

## Supplementary Information

Additional file 1: Fig. S1. Protein sequence alignment of *Bos taurus* BMAL1 and *Bos taurus* HIF1β (ARNT).

Additional file 2: Fig. S2. Protein sequence alignment of *Bos taurus* CLOCK and *Bos taurus* HIF1α.

Additional file 3: Table S1. Power analysis.

Additional file 4: Table S2. qPCR primers and siRNA sequence.

Additional file 5: Table S3. 15 mmol/L sodium lactate reprograms mammary gland diurnal transcriptome.

Additional file 6: Table S4. 30 mmol/L sodium lactate reprograms mammary gland diurnal transcriptome.

Additional file 7: Table S5. Sequences of primers for ChIP-qPCR analysis.

## Data Availability

The raw sequencing datasets generated in this study were submitted to the Genome Sequence Archive (GSA) with the identifier CRA019241.

## Abbreviations

bHLH-PAS: Basic helix-loop-helix PER-ARNT-SIM
DEGs: Differential expression genes
HIF1α: Hypoxia inducible factor 1 subunit alpha
HREs: Hypoxia-response elements
GSH-Px: Glutathione peroxidase
GPR81: G protein-coupled receptor 81
MDA: Malondialdehyde
MCTs: Monocarboxylate transporters
ROS: Reactive oxygen species
SCC: Somatic cell count
SOD: Superoxide dismutase
T-AOC: Total antioxidant capacity
T-NOS: Total nitric oxide synthase
TFs: Transcription factors
VEGF: Vascular endothelial growth factor
VHL: Von Hippel-Lindau

## References

[1] Pundir CS, Narwal V, Batra B. Determination of lactic acid with special emphasis on biosensing methods: A review. Biosens Bioelectron. 2016;86:777–90. 27476060 10.1016/j.bios.2016.07.076

[2] Brooks GA. Lactate as a fulcrum of metabolism. Redox Biol. 2020;35:101454. 32113910 10.1016/j.redox.2020.101454 PMC7284908

[3] Khatib-Massalha E, Bhattacharya S, Massalha H, Biram A, Golan K, Kollet O, et al. Lactate released by inflammatory bone marrow neutrophils induces their mobilization via endothelial GPR81 signaling. Nat Commun. 2020;11:3547. 32669546 10.1038/s41467-020-17402-2 PMC7363928

[4] Felmlee MA, Jones RS, Rodriguez-Cruz V, Follman KE, Morris ME. Monocarboxylate transporters (SLC16): function, regulation, and role in health and disease. Pharmacol Rev. 2020;72(2):466–85. 32144120 10.1124/pr.119.018762 PMC7062045

[5] Brooks GA. The science and translation of lactate shuttle theory. Cell Metab. 2018;27(4):757–85. 29617642 10.1016/j.cmet.2018.03.008

[6] Semenza GL. Hypoxia-inducible factor 1 (HIF-1) pathway. Science’s STKE. 2007;2007:cm8. 10.1126/stke.4072007cm8 17925579

[7] Sonveaux P, Copetti T, De Saedeleer CJ, Végran F, Verrax J, Kennedy KM, et al. Targeting the lactate transporter MCT1 in endothelial cells inhibits lactate-induced HIF-1 activation and tumor angiogenesis. PLoS ONE. 2012;7(3):e33418. 22428047 10.1371/journal.pone.0033418 PMC3302812

[8] Sanmarco LM, Rone JM, Polonio CM, Fernandez Lahore G, Giovannoni F, Ferrara K, et al. Lactate limits CNS autoimmunity by stabilizing HIF-1α in dendritic cells. Nature. 2023;620(7975):881–9. 37558878 10.1038/s41586-023-06409-6 PMC10725186

[9] Semenza GL. Hypoxia-inducible factors in physiology and medicine. Cell. 2012;148:399–408. 22304911 10.1016/j.cell.2012.01.021 PMC3437543

[10] Bass J, Lazar MA. Circadian time signatures of fitness and disease. Science. 2016;354(6315):994–9. 27885004 10.1126/science.aah4965

[11] Rey G, Reddy AB. Connecting cellular metabolism to circadian clocks. Trends Cell Biol. 2013;23(5):234–41. 23391694 10.1016/j.tcb.2013.01.003

[12] Bass J. Circadian topology of metabolism. Nature. 2012;491:348–56. 23151577 10.1038/nature11704

[13] Bass J, Takahashi JS. Circadian integration of metabolism and energetics. Science. 2010;330(6009):1349–54. 21127246 10.1126/science.1195027 PMC3756146

[14] Quist MA, LeBlanc SJ, Hand KJ, Lazenby D, Miglior F, Kelton DF. Milking-to-milking variability for milk yield, fat and protein percentage, and somatic cell count. J Dairy Sci. 2008;91(9):3412–23. 18765600 10.3168/jds.2007-0184

[15] Wang M, Zhou Z, Khan MJ, Gao J, Loor JJ. Clock circadian regulator (CLOCK) gene network expression patterns in bovine adipose, liver, and mammary gland at 3 time points during the transition from pregnancy into lactation. J Dairy Sci. 2015;98(7):4601–12. 25912864 10.3168/jds.2015-9430

[16] Budkowska M, Cecerska-Heryć E, Marcinowska Z, Siennicka A, Dołęgowska B. The influence of circadian rhythm on the activity of oxidative stress enzymes. Int J Mol Sci. 2022;23(22):14275. 36430753 10.3390/ijms232214275 PMC9697911

[17] National Academies of Sciences, Engineering, and Medicine. Nutrient requirements of dairy cattle: 8th re ed. Washington, DC: The National Academies Press; 2021. 38386771

[18] Zhang X, Wang D, Liu J. Hypoxia-inducible factor-1α is involved in the response to heat stress in lactating dairy cows. J Therm Biol. 2023;112:103460. 36796905 10.1016/j.jtherbio.2023.103460

[19] Cai J, Peng J, Feng J, Li R, Ren P, Zang X, et al. Antioxidant hepatic lipid metabolism can be promoted by orally administered inorganic nanoparticles. Nat Commun. 2023;14:3643. 37339977 10.1038/s41467-023-39423-3 PMC10281969

[20] Liao Y, Wang J, Jaehnig EJ, Shi Z, Zhang B. WebGestalt 2019: gene set analysis toolkit with revamped UIs and APIs. Nucleic Acids Res. 2019;47:W199–205. 31114916 10.1093/nar/gkz401 PMC6602449

[21] Levine DC, Hong H, Weidemann BJ, Ramsey KM, Affinati AH, Schmidt MS, et al. NAD^+^ controls circadian reprogramming through PER2 nuclear translocation to counter aging. Mol Cell. 2020;78(5):835-849.e7. 32369735 10.1016/j.molcel.2020.04.010 PMC7275919

[22] Cai J, Wang D, Liang S, Peng J, Zhao F, Liu J. Excessive supply of glucose elicits an NF-κB2-dependent glycolysis in lactating goat mammary glands. FASEB J. 2020;34(6):8671–85. 32359096 10.1096/fj.201903088R

[23] Tauffenberger A, Fiumelli H, Almustafa S, Magistretti PJ. Lactate and pyruvate promote oxidative stress resistance through hormetic ROS signaling. Cell Death Dis. 2019;10(9):653. 31506428 10.1038/s41419-019-1877-6 PMC6737085

[24] Lu H, Forbes RA, Verma A. Hypoxia-inducible factor 1 activation by aerobic glycolysis implicates the Warburg effect in carcinogenesis. J Biol Chem. 2002;277(26):23111–5. 11943784 10.1074/jbc.M202487200

[25] Tan Z, Xie N, Banerjee S, Cui H, Fu M, Thannickal VJ, et al. The monocarboxylate transporter 4 is required for glycolytic reprogramming and inflammatory response in macrophages. J Biol Chem. 2015;290:46–55. 25406319 10.1074/jbc.M114.603589 PMC4281748

[26] Maxwell PH, Pugh CW, Ratcliffe PJ. Activation of the HIF pathway in cancer. Curr Opin Genet Dev. 2001;11(3):293–9. 11377966 10.1016/s0959-437x(00)00193-3

[27] Semenza GL. HIF-1: mediator of physiological and pathophysiological responses to hypoxia. J Appl Physiol (1985). 2000;88(4):1474–1480. 10.1152/jappl.2000.88.4.1474 10749844

[28] Masri S, Sassone-Corsi P. The emerging link between cancer, metabolism, and circadian rhythms. Nat Med. 2018;24(12):1795–803. 30523327 10.1038/s41591-018-0271-8 PMC6535395

[29] Panda S. Circadian physiology of metabolism. Science. 2016;354(6315):1008–15. 27885007 10.1126/science.aah4967 PMC7261592

[30] Kim Y, Nam HJ, Lee J, Park DY, Kim C, Yu YS, et al. Methylation-dependent regulation of HIF-1α stability restricts retinal and tumour angiogenesis. Nat Commun. 2016;7:10347. 26757928 10.1038/ncomms10347 PMC4735525

[31] Peek CB, Levine DC, Cedernaes J, Taguchi A, Kobayashi Y, Tsai SJ, et al. Circadian clock interaction with HIF1α mediates oxygenic metabolism and anaerobic glycolysis in skeletal muscle. Cell Metab. 2017;25(1):86–92. 27773696 10.1016/j.cmet.2016.09.010 PMC5226863

[32] Marri D, Filipovic D, Kana O, Tischkau S, Bhattacharya S. Prediction of mammalian tissue-specific CLOCK-BMAL1 binding to E-box DNA motifs. Sci Rep. 2023;13:7742. 37173345 10.1038/s41598-023-34115-w PMC10182026

[33] McIntosh BE, Hogenesch JB, Bradfield CA. Mammalian Per-Arnt-Sim proteins in environmental adaptation. Annu Rev Physiol. 2010;72:625–45. 20148691 10.1146/annurev-physiol-021909-135922

[34] Bersten DC, Sullivan AE, Peet DJ, Whitelaw ML. bHLH-PAS proteins in cancer. Nat Rev Cancer. 2013;13(12):827–41. 24263188 10.1038/nrc3621

