# Supplementary material for: Lactate induces oxidative stress by HIF1α stabilization and circadian clock disturbance in mammary gland of dairy cows
Source: J Anim Sci Biotechnol. 2025 May 1;16:62. doi: 10.1186/s40104-025-01181-1 (PMC12044779; doi:10.1186/s40104-025-01181-1)
Supplement: Supplementary file 1 — Additional file 1: Fig. S1. Protein sequence alignment of Bos taurus BMAL1 and Bos taurus HIF1β (ARNT). [file 40104_2025_1181_MOESM1_ESM.docx]

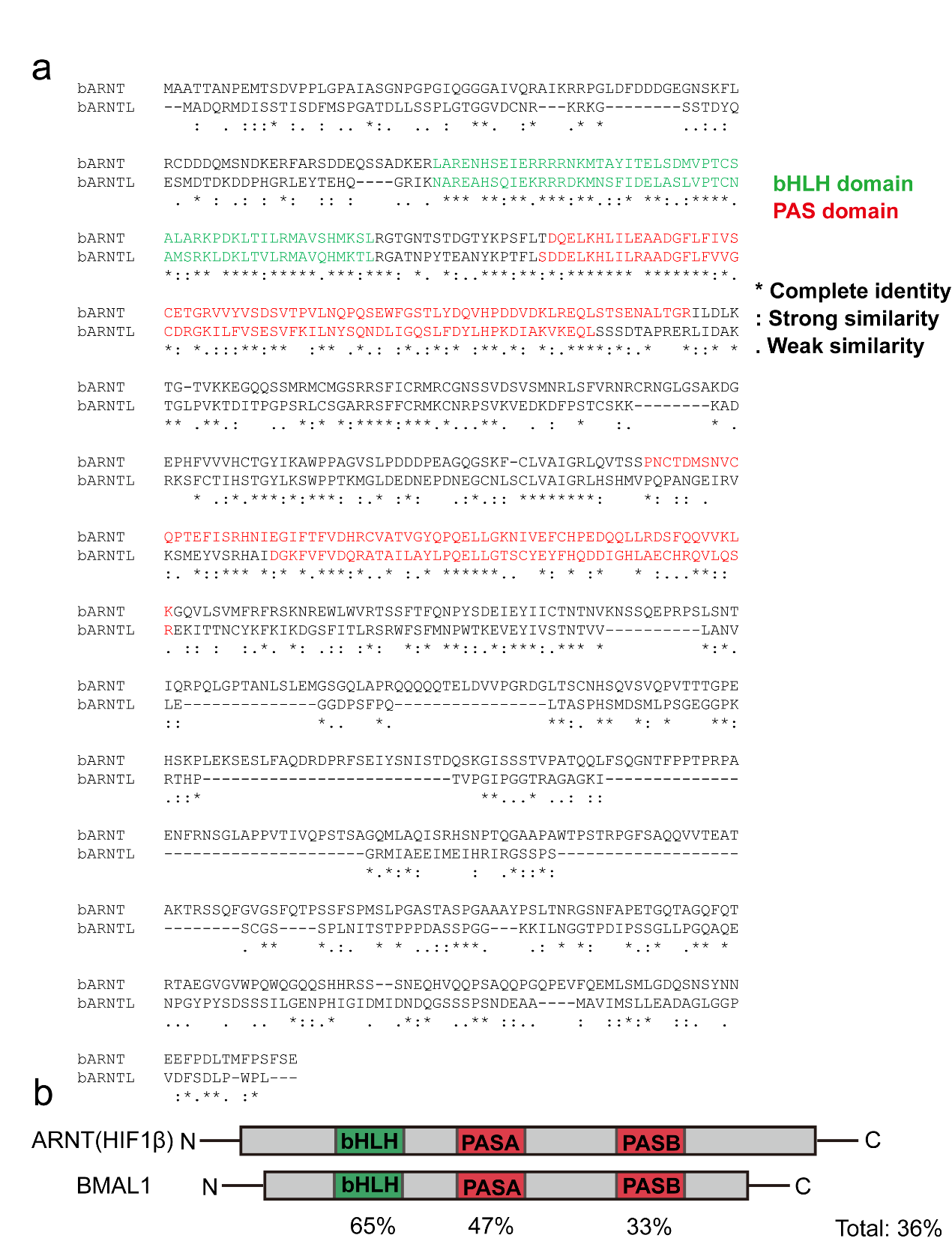


**Fig. S1.** Protein sequence alignment of Bos taurus BMAL1 and Bos taurus HIF1β (ARNT). (a) Full-protein sequences were aligned using Multiple Sequence Comparison by Log-Expectation (MUSCLE) software available through the European Bioinformatics Institute (EMBL; http://www.ebi.ac.uk) (Edgar, 2004b) (Edgar, 2004a). bHLH and PAS domains are highlighted. Consensus symbols indicate complete identity (*), strong similarity (scoring >0.5 in the Gonnet PAM 250 matrix) (:), and weak similarity (scoring <0.5 in the Gonnet PAM 250 matrix) (.) (http://www.ebi.ac.uk/Tools/msa/clustalw2/help/index.html). (b) Schematic of BMAL1 and HIF1β (ARNT) proteins with % identity in the bHLH and PAS domains regions (not to scale).
