# Supplementary material for: Lactate induces oxidative stress by HIF1α stabilization and circadian clock disturbance in mammary gland of dairy cows
Source: J Anim Sci Biotechnol. 2025 May 1;16:62. doi: 10.1186/s40104-025-01181-1 (PMC12044779; doi:10.1186/s40104-025-01181-1)
Supplement: Supplementary file 3 — Additional file 3: Table S1. Power analysis. [file 40104_2025_1181_MOESM3_ESM.docx]

**Table S1.** Power analysis

| **Index** | **Group** | **Power** |
| --- | --- | --- |
| 1 | 2 | 0.178 |
| 2 | 3 | 0.366 |
| 3 | 4 | 0.534 |
| 4 | 5 | 0.667 |
| 5 | 6 | 0.768 |
| 6 | 7 | 0.841 |
| 7 | 8 | 0.893 |
| 8 | 9 | 0.929 |
| 9 | 10 | 0.954 |
| 10 | 12 | 0.981 |
| 11 | 14 | 0.992 |
| 12 | 16 | 0.997 |
| 13 | 18 | 0.999 |
| 14 | 20 | >.999 |
| 15 | 25 | >.999 |
| 16 | 30 | >.999 |
| 17 | 35 | >.999 |
| 18 | 40 | >.999 |
| 19 | 45 | >.999 |
| 20 | 50 | >.999 |
