# Supplementary material for: Lactate induces oxidative stress by HIF1α stabilization and circadian clock disturbance in mammary gland of dairy cows
Source: J Anim Sci Biotechnol. 2025 May 1;16:62. doi: 10.1186/s40104-025-01181-1 (PMC12044779; doi:10.1186/s40104-025-01181-1)
Supplement: Supplementary file 4 — Additional file 4: Table S2. qPCR primers and siRNA sequence. [file 40104_2025_1181_MOESM4_ESM.docx]

**Table S2**. qPCR primers and siRNA sequence

| **qPCR primers** | **Sequence** |
| --- | --- |
| Cow *HIF1A* Forward | CAGAGCAGGAAAGAGAGTCATAGAAC |
| Cow *HIF1A* Reverse | TTTCGCTT CCTCTGAGCATTC |
| Cow Gapdh Forward | GGGACCTGACTGACTACCTC |
| Cow Gapdh Reverse | TCATACTCCTGCTTGCTGAT |
| **siRNA** | **Target sequence** |
| siNC | GCACTACCAGAGCTAACTCA |
| siHIF1α | AGATGCGAACTCACATTATG |
