# Supplementary material for: Lactate induces oxidative stress by HIF1α stabilization and circadian clock disturbance in mammary gland of dairy cows
Source: J Anim Sci Biotechnol. 2025 May 1;16:62. doi: 10.1186/s40104-025-01181-1 (PMC12044779; doi:10.1186/s40104-025-01181-1)
Supplement: Supplementary file 7 — Additional file 7: Table S5. Sequences of primers for ChIP-qPCR analysis. [file 40104_2025_1181_MOESM7_ESM.docx]

**Table S5**. Sequences of primers for ChIP-qPCR analysis

| **Name** | **Forward (5'-3'sequence)** | **Reverse (5'-3'sequence)** |
| --- | --- | --- |
| cBMAL1 | TTCCTTGCAGCTCTCAGTCA | CGATACTACCAGCACCGAGT |
| cHMOX1 | AAGATTGCTCAGAAGGCCCT | ACAGCTGCTTGAACTTGGTG |
| cFOXO3 | CTCAACCAGTGCGAACCTTC | CCAACCCATCAGCATCCATG |

c: cow
